# LNDb v4: pulmonary nodule annotation from medical reports

**DOI:** 10.1038/s41597-024-03345-6

**Published:** 2024-05-17

**Authors:** Carlos A. Ferreira, Célia Sousa, Inês Dias Marques, Pedro Sousa, Isabel Ramos, Miguel Coimbra, Aurélio Campilho

**Affiliations:** 1 Institute for Systems and Computer Engineering, Technology and Science (INESC TEC), Porto, Portugal; 2 Faculty of Engineering of the University of Porto (FEUP), Porto, Portugal; 3 Department of Radiology, Unidade Local de Saúde de Gaia/Espinho (ULSGE), Porto, Portugal; 4 Department of Radiology, Centro Hospitalar Universitário de São João (CHUSJ), Porto, Portugal; 5 Faculty of Medicine of the University of Porto (FMUP), Porto, Portugal; 6 Faculty of Sciences of the University of Porto (FCUP), Porto, Portugal

**Keywords:** Lung cancer, Computed tomography

## Abstract

Given the high prevalence of lung cancer, an accurate diagnosis is crucial. In the diagnosis process, radiologists play an important role by examining numerous radiology exams to identify different types of nodules. To aid the clinicians’ analytical efforts, computer-aided diagnosis can streamline the process of identifying pulmonary nodules. For this purpose, medical reports can serve as valuable sources for automatically retrieving image annotations. Our study focused on converting medical reports into nodule annotations, matching textual information with manually annotated data from the Lung Nodule Database (LNDb)—a comprehensive repository of lung scans and nodule annotations. As a result of this study, we have released a tabular data file containing information from 292 medical reports in the LNDb, along with files detailing nodule characteristics and corresponding matches to the manually annotated data. The objective is to enable further research studies in lung cancer by bridging the gap between existing reports and additional manual annotations that may be collected, thereby fostering discussions about the advantages and disadvantages between these two data types.

## Background & Summary

Lung cancer ranks among the most frequently diagnosed cancers and holds the highest mortality rate among all types of cancer^1^. This type of cancer is strongly associated with heavy smoking habits, and when symptoms are present or detected in screening programs, Computed Tomography (CT) is used for the initial diagnosis.

Radiologists play a pivotal role in lung cancer diagnosis as they meticulously examine and annotate CTs. Upon receiving the exams, radiologists identify pathological findings, including lung nodules or masses, which can manifest diverse shapes and appearances within lung tissue and serve as crucial indicators of cancer. Nevertheless, the search for nodules is an arduous, error-prone, and time-consuming process due to the intricate three-dimensional nature of CTs. Furthermore, nodules exhibit distinct textures: solid, part-solid, and Ground-Glass Opacitys (GGOs), adding complexity to the detection process. These textural variations, along with the similarity of nodules to other anatomical structures, contribute to scanning errors ( ~ 30%), recognition inaccuracies (~25%), and decision-making difficulties (~45%)^2^. To address these issues and provide additional diagnostic support, Artificial Intelligence (AI) solutions are being considered for integration into clinical practice. However, the systematic adoption of such solutions encounters obstacles related to data quantity, representativeness, and the extensive annotation required for algorithm training.

Medical practice gives us an opportunity to gather more data to train AI algorithms, but observed findings are typically documented only in written medical reports, and not conveniently as marked spatial regions of interest associated with the images themselves. This is understandable since for clinicians, free text is an easy way to record and access medically relevant information. Having access to the marked spatial regions of interest would not only benefit the training of AI algorithms, but also other healthcare professionals who need to review these reports at a later stage, who may find this information incomplete or inconsistent^3^. If a radiology report omits a key finding or fails to effectively communicate the importance of a reported finding, subsequent clinical decisions regarding management or future follow-up may be affected^4,5^.

For future reports, we could promote the marking of these spatial regions of interest using specialized software, which however comes with a higher temporal cost for the creation of this report, the need to learn and use specialized software, and does not solve the problem of past reports. As an alternative, we could explore methods for extracting annotations from medical reports using text mining, followed by the mapping of this natural language information into spatial regions of interest of associated images. Today, for the purposes of AI training, we can find fully labeled datasets from medical reports, such as CheXpert^6^ and DeepLesion^7^, as well as image datasets with manual annotation by radiologists, such as LIDC-IDRI^8^.

In 2019, the Lung Nodule Database (LNDb) database was released^9^, comprising chest CT images with manual nodule segmentation and texture characterization. This manuscript describes additional nodule characterizations, including calcification, internal structure, lobulation, malignancy, margin, sphericity, spiculation, and subtlety. Additionally, medical reports for the same exams were collected as part of this study. The information related to the nodules in these medical reports has been gathered, structured, and described here to share with the community. The information from these reports was compared to the manually annotated findings to assess the match and mismatches between the two data sources. To the best of our knowledge, this is the first time where manual and report-based annotations have been made available and their correspondence has been analyzed, particularly in the context of CT scans. The aim of sharing this data is to encourage the community to consider the reuse of data, such as medical reports, and to highlight the accuracy, consistency, and potential biases associated with each annotation source.

## Methods

The LNDb database was developed within the LNDetector project (Project funded by the Portuguese funding agency, FCT - Fundação para a Ciência e a Tecnologia). The project aimed to create a comprehensive lung cancer screening system, incorporating modules for for AI-based detection, segmentation, classification, and nodule follow-up to aid in pulmonary cancer management. Throughout the clinical study for data collection, hospital protocols remained unchanged. Radiologists and various clinicians involved in the process performed their routine clinical practice. A CT scan was deemed eligible if the slice thickness was 1 mm.

All data acquisition procedures were conducted in compliance with the approval granted by the CHUSJ Ethical Committee (PTDC/EEI-SII/6599/2014, POCI-01-0145-FEDER-016673). Participants did provide informed consent for the open publication of their data. It should be noted that data collection was done prospectively, with a guarantee of anonymization. Among the 294 patients scanned, 164 (55.8%) were male. The median age was 66 and the minimum and maximum ages were 19 and 98, respectively.

During the medical reporting process, radiologists routinely described all thoracic findings without specifying spatial coordinates or considering potential inclusion in a clinical study. The exact number of radiologists participating in text reporting is unknown. For the manual annotation process, five radiologists were tasked with annotating nodular findings. Each CT scan received between one and three annotations from different radiologists. Guidelines also required the annotation of non-nodules, which are findings closely resembling nodules but not actual nodules themselves.

Upon the release of the dataset, the LNDb Grand Challenge was launched (https://lndb.grand-challenge.org/)^10^, encouraging the community to propose solutions for the nodule detection, nodule segmentation, texture classification, and follow-up recommendation according to Fleischner guidelines^11^. The imaging data and manual annotations were described in Pedrosa *et al*.^12^ and stored on the Zenodo platform^9^. Contributions for evaluating results on the test set are still being accepted.

In order to enable broader usage of the dataset, the remaining characteristics of lung nodules annotated by the radiologists in this study, namely calcification, internal structure, lobulation, malignancy, margin, sphericity, spiculation, and subtlety, are also being released and described in this manuscript.

Nonetheless, the main objective of the manuscript is to release the description of lung nodules from medical reports into a tabular format, while identifying their spatial locations through cross-referencing with manually annotated data^13^. As there is no definitive ground truth due to the temporal gap between the textual reporting and the manual annotations, they do not influence each other. Specific criteria were established, as detailed in this section, to optimize the identification of matches between the nodules described in the reports and those delineated in the image annotations. As a result, when a correlation is established, the spatial coordinates were obtained.

In order to release the structured descriptions of lung nodules mentioned in the medical reports and evaluate their match with the previously released manual annotations, a methodology comprising three phases was established:

- **Medical Reports Annotation**: how the annotator described the detected nodules;
- **Tabular Data Conversion**: explaining the process of obtaining the structured file;
- **Nodule Matching**: outlining the criteria used to determine the match.

**Fig. 1:**
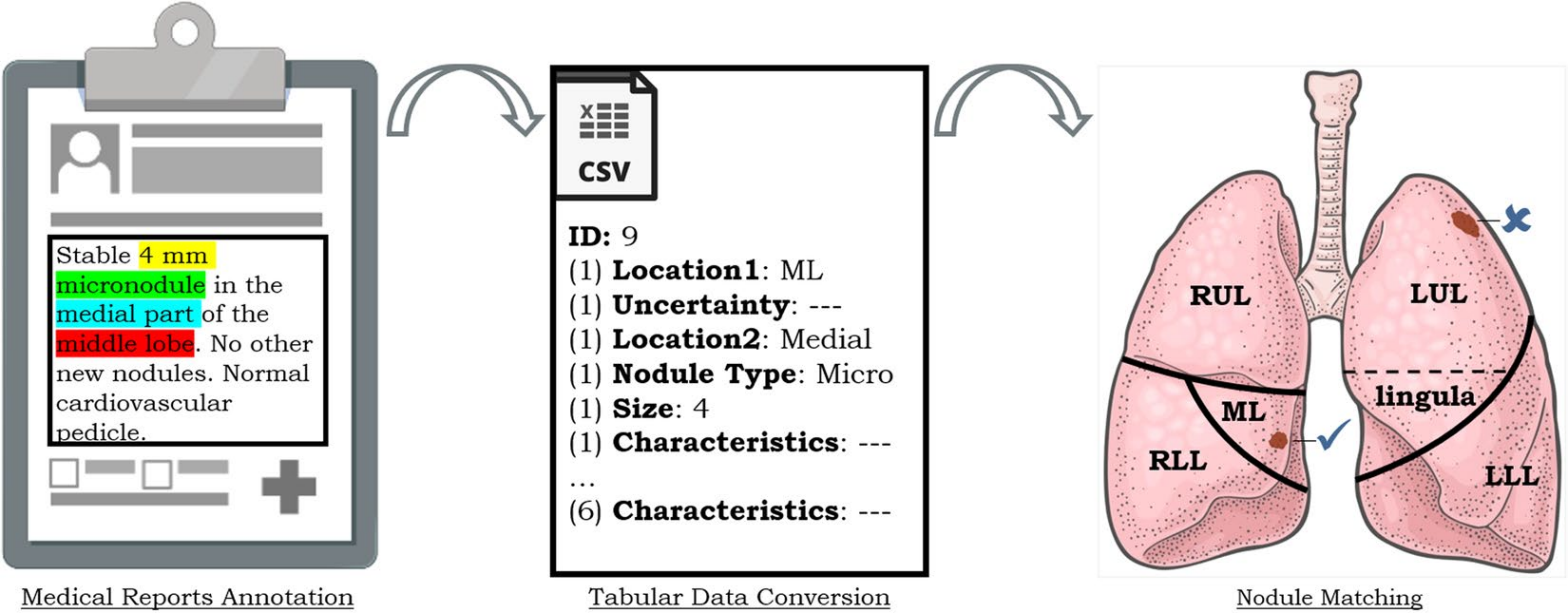
Schematic overview of the assay phases in this study: i) Initially, all information related to the lung nodule is highlighted in the medical report; ii) The pieces of text are converted to their respective fields in the entities table; iii) The correspondence is verified between the two nodules that had been manually annotated, and only one of them corresponds to the ML in terms of relative position and written characteristics, given by ✓, unlike the other one, given by ✗.

Figure 1 provides a schematic representation of the phases with illustrative images for each one, which is further described in the following subsections. Different colors are used in the report to highlight various attributes. The central image illustrates how this information translates into tabular terms, while the lung image demonstrates the nodule chosen among the possibilities based on the written description.

### Medical Reports Annotation & Tabular Data Conversion

The medical reports were provided in Portuguese, the official language of the partner hospital, and were anonymized. The reports consist of a single section that describes all observations related to atypical landmarks found in thoracic organs during the CT scans, including conditions such as cardiomegaly, emphysema, and effusions, among others. However, the reports do not specifically focus on nodules. We use the term ‘entity’ to refer to text portions describing a nodule or set of nodules with shared characteristics. Following an analysis phase and the identification of text regions pertaining to the nodules, it was observed that the entities may contain some of the following attributes:

- **Location1**: Right Upper Lobe (RUL), Middle Lobe (ML), Right Lower Lobe (RLL), Left Upper Lobe (LUL), Left Lower Lobe (LLL) upper lobes, lower lobes, right lung, left lung, lingula, and lingula/LLL.
  The first five are the lobes, which are often represented as acronyms in the literature. The lingula is a part of the LUL, and the remaining ones consist of a collection of various lobes, each designated accordingly. Figure 1 offers a schematic representation of the lung lobes.
- **Location2**: apical/superior, basal/inferior, anterior, posterior, medial, lateral, centrilobular, and justapleural/peripheral.
  These references indicate relative positions. It is possible that more than one of these options may be mentioned for the same entity. The first three pairs refer to the axial, coronal, and sagittal planes, respectively. The last pair indicates the position within the lobe.
- **Uncertainty**: “how many?” and “is it?”.
  In this case, there was a need to convert and interpret the text. These situations are considered when there is uncertainty about the quantity of nodules mentioned in a sentence. The first case applies when there are nodular references in the plural form, while the second case applies when words indicating probability, such as “probably” or “maybe”, are used with the description of the nodules. Both expressions of uncertainty can be found simultaneously for the same entity.
- **Lesion Type**: micronodule, nodule, mass and granuloma (*i.e*., nodules with calcifications).
- **Size**: numerical value indicating the diameter.
  If the volume is provided, and considering the typical shape of the lesions, the diameter is considered as the equivalent diameter of a sphere. If multiple measurements are indicated for the size axes of the nodule, the average is taken.
- **Characteristics**: texture, calcification, internal structure, lobulation, malignancy, margin, sphericity, spiculation and subtlety.
  The characteristics are interpreted based on the information provided in the text, following the characterization table for LNDb and LIDC-IDRI^14^, as shown in Table 1. All characteristics are ranked continuously from 1 to 5, except for categorical variables like internal structure, which has 4 rankings, and calcification, which has 6 rankings.

**Table 1:** Nodule characteristics and meaning of 1-6 classes for each characteristic as defined on LNDb annotations.

Nodule Feature	1	2	3	4	5	6
Internal Structure	Soft tissue	Fluid	Fat	Air	—	—
Texture	Non-solid	...	Part solid	...	Solid	—
Subtlety	Extremely subtle	Very subtle	Subtle	Relatively obvious	Obvious	—
Calcification	Popcorn	Laminated	Solid	Non-central	Central	Absent
Sphericity	Linear	...	Ovoid	...	Round	—
Margin	Poorly defined	...	...	...	Sharp	—
Lobulation	No lobulation	...	...	...	Marked	—
Spiculation	No spiculation	...	...	...	Marked	—
Malignancy	Highly unlikely	Moderately unlikely	Indeterminate	Moderately suspicious	Highly suspicious	—

... represents an intermediate characteristic compared to what is written on that row and - indicates the absence of a numerical assignment for that characteristic.

The annotation and conversion of the data into a tabular format were conducted by an analyst with over 5 years of experience in handling lung cancer CT data. Validation was sought from an expert to address any uncertainties that arose during the process.

### Nodule matching

**Fig. 2:**
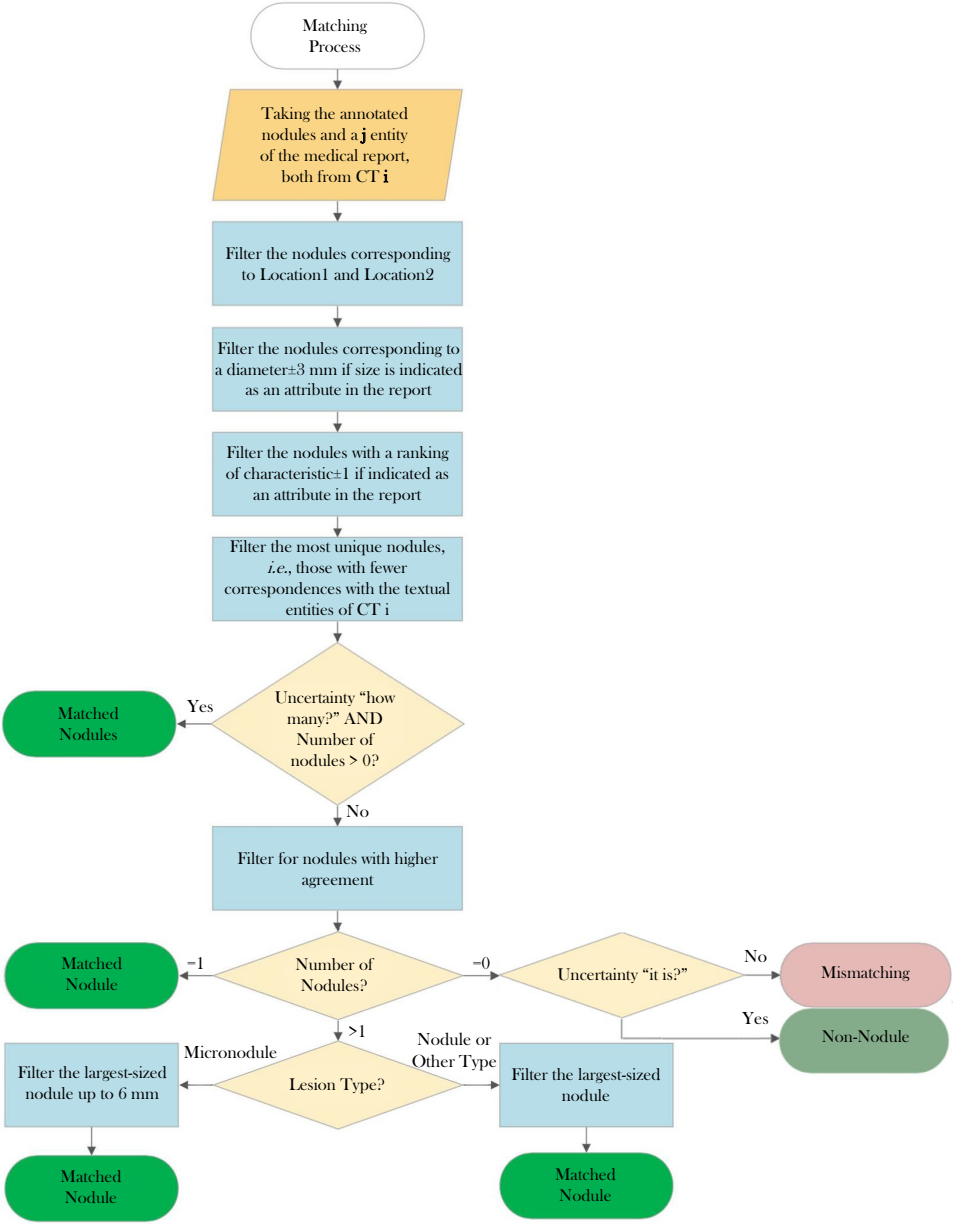
Summarized flowchart encompassing the various criteria used to establish the nodule matching process.

The matching process involves two phases: candidate search and matching decision. A flowchart in Fig. 2 illustrates the process. During the candidate search, each entity in a medical report is matched with manually annotated nodules based on specific criteria. If any of the criteria is absent in the medical report during the candidate search phase, only the remaining ones are checked. The criteria are as follows:

- **Location1**: The spatial location is available in the manual annotation. However, it is necessary to identify the lobe corresponding to the annotated nodules because the reports mention the lobe rather than the spatial coordinates. For this, an automated method was employed, using the algorithm developed by Hofmanninger *et al*.^15^ for lobe segmentation. It is the publicly available method with the best state-of-the-art results. This method employed a 2D UNet^16^ trained on a slice-by-slice basis with diverse datasets covering different diseases. Achieving a Dice similarity coefficient of 0.97, they demonstrated that automatic lobe segmentation is more influenced by data diversity than methodological limitations. Hence, employing Hofmanninger *et al*.’s algorithm enables the conversion of nodule centroid coordinates to one of the 5 lobes. When a nodule was indicated to be in the lingula, all nodules in the LUL that were lower to the upper coordinate of the LLL were considered.
- **Location2**: The centroid of the lobe served as the reference point. This facilitated verifying the relative position of the nodule’s centroid in relation to the lobe’s centroid, confirming the position along the three anatomical axes. For nodules described as peripheral, their position was checked within a quarter of each anatomical axis’s size within the lobe, adjacent to lung walls. Nodules described as centrilobular were considered to be located in the central region of the lung, with the lobe region extending up to half the size of the anatomical axes;
- **Size**: The diameter reported in the tabular data conversion was compared with the equivalent diameter of each annotated nodule for the same CT scan. A tolerance of ± 3 mm was applied in the size comparison due to uncertainty regarding whether the reported size consistently referred to the equivalent diameter, an approximate measurement, or the major or minor axis of a nodule;
- **Characteristics**: Correspondence between the numerical values in the tabular data and those assigned by radiologists in manual annotation was assessed. A tolerance of ±  1 was allowed for inclusion as a candidate.

In the matching decision phase, before evaluating each criterion, the number of candidates is verified. If a previously annotated nodule has already been matched with an entity from the same CT scan, it is removed from the pool of candidates. If no nodules remain, it is considered a mismatch unless the uncertainty criterion “is it?” is present. If there is more than one candidate, the next criterion is considered. If only one candidate remains, the match is considered found. The matching criteria were as follows:

- **Uniqueness**: Among the pool of annotated nodules, only those that appear less frequently as candidates for different entities are considered;
- **Agreement**: This criterion selects the findings that have a higher number of radiologists as annotators;
- **Size + Lesion Type**: If the size is indicated in the medical report, the annotated nodule with the closest size is chosen. If the size is not indicated, the largest nodule is selected. If the nodule is referred to as a micronodule, it is considered to have a maximum size of up to 6 mm. It was observed that micronodules are often reported with sizes up to 6 mm in these reports, even though officially a micronodule is considered to be up to 3 mm. Therefore, a limit of 6 mm was considered.

If the uncertainty parameter “how many?” is present, the process stops at the first criterion. Otherwise, all criteria must be passed and fulfilled. Ties may occur in the first and second matching criteria in some cases, while the decision in the last criterion is made unequivocally based on the considerations. Considering uniqueness, agreement, and size in the matching decision is deemed reasonable, and it is believed that these considerations do not restrict the potential evidences from this study.

## Data Records

The data structure in Zenodo^13^ is presented first. The LNDb dataset comprises 294 CT scans collected between 2016 and 2018, available in MetaImage (*.mhd*/*.raw*) format. Filenames follow the pattern *LNDb-XXXX.mhd*, where *XXXX* is the LNDb CT Identification (ID). This image data was divided into 4 GB archives (*data0.rar*, *data1.rar*, *data2.rar*, *data3.rar*, *data4.rar*, *data5.rar*, *testdata0.rar*, and *testdata1.rar*). In these archives, we can also find the image IDs for training and testing respectively in *trainCTs.csv* and *testCTs.csv*. Acquisition parameters for each CT are in *LNDbAcqParams.csv*.

In *trainset_csv.zip*, we have the files *trainFolds.csv* (with the CT list in each train/validation fold), *trainNodules.csv* (with nodule annotation for training/validation as annotated by radiologist, containing texture characterization), *trainNodules_gt.csv* (with nodule annotation for training/validation after merging annotations by different radiologists), and *trainFleischner.csv* (with the Fleischner scores considering only manual annotation in the the training/validation phase).

Nodule segmentations are provided in MetaImage (*.mhd*/*.raw*) format. Each *LNDbXXXX_radR.mhd* file contains the segmentation for all nodules in the CT *XXXX* according to radiologist *R*, stored in a *3D* array of the CT’s size, where each pixel represents the finding’s ID in *trainNodules.csv*.

The nodules in LNDb medical reports, together with the nodule spatial positions (when available), are released with this article. For this purpose, 6 new files are in Zenodo^13^.

The file *report.csv* contains tabular data extracted from the medical reports of the CT scans in the dataset. The generation of this tabular file is described in the “Medical Reports Annotation & Tabular Data Conversion” of the “Methods” section.

In the matching phase, the characteristics^17^ of manually annotated nodules were used. This data is available in the file *chars_trainNodules.csv*.

After the matching process, three files are generated: *rad2Fleischner.csv*, *text2Fleischner.csv*, and *allNods.csv*. The first file contains a list of image-annotated nodules, while the second file contains the nodules reported in the medical reports. The last file, *allNods.csv*, includes all nodules identified in the study. For each nodule, the files provide information about whether it was manually annotated, reported textually, or both, along with its location and characteristic attributes.

## Technical Validation

In this section, we describe some experiments to assess the validity of the method outlined in this article. For this purpose, the technical validation was divided into two parts: clinical assessment and data insights. In the clinical assessment, a radiologist was asked to evaluate the matching between the medical reports and the manual annotations in the test set. The clinical perspective serves as confirmation that a radiologist is able to identify the same nodules, identified by the method described here, using only the textual information in the clinical reports. In the data insights, we analyze the data to verify if the obtained matching correspond to what is described in recently published research.

### Clinical assessment

In the clinical assessment, a radiologist with 7 years of experience, who had not previously participated in the study, was invited to determine if he would establish the same correspondence between an entity in a medical report and a manually annotated nodule. This validation followed the following steps:

- Initially, a file containing the sentences from the medical reports of each nodule and their respective centroids was provided to this radiologist;
- The radiologist was asked to classify each matching as *correct*, *imprecise*, or *incorrect*. *Correct* means that the nodule was accurately matched with its corresponding entity in the medical report. *Imprecise* indicates that the nodule was matched, but there were other nodules with similar characteristics that could also have been matched. *Incorrect* signifies that the nodule was wrongly matched with an entity in the medical report, showing no correlation between the two;
- The radiologist viewed and classified all matching cases for each exam using a CT image visualization software^18^.

In 81.3% of cases, the result was *correct* or *imprecise*. The 7.5% considered *imprecise* correspond to nodules whose calcification classifications are ambiguous. Regarding those where there was an *incorrect* decision, the radiologist in the study considered that:

- 5.0% corresponded to a scar in the manual annotation;
- 5.0% had a different location, within a fissure, which was not evaluated in this study;
- 3.8% were identified as a different type of nodule;
- 2.5% had a different size than reported;
- There was one case where the correspondences between phrases and two nodules were swapped.

It is uncertain to what extent these results may have been influenced by the lack of systematicity in textual reporting or differing considerations among clinicians. The radiologist in this study identified some matches as scars, which were not expected in the annotation. Different sizes and types of lesions may also stem from differing considerations among the radiologists initially involved in textual reporting and manual annotation compared to the new radiologist in this context.

### Data insights

As discussed in the background section, published research indicates that medical reports are more objective, whereas manual image annotation in dedicated studies tends to lead to over-annotation including irrelevant findings^19–21^. Nevertheless, it is uncommon that these differences lead to significant discrepancies in diagnosis^22,23^. In this subsection, we aim to verify if the evidence in this study aligns with what is reported in the literature.

In medical reports, matching is complete in 58.9%, and only one entity remains unmatched in 30.7%. Complete means that all entities mentioned in the medical reports had matches. In manual image annotation, the correspondence with all nodules in the medical reports was achieved for 26.0% of cases, while in 25.3% there is no overlap at all between the entities of medical reports and manual annotations. This result is consistent with the literature^19–21^, meaning that manual annotations tend to be more numerous when compared to findings in medical reports.

The differences in the number of matched and mismatched nodules is listed in Table 2, presenting various diameter ranges and the number of nodules in each category. No significant differences in nodule types were evident, except for a discrepancy in the percentage of nodules smaller than 3 mm in manual annotations, likely not warranting textual relevance. As for the characteristics of nodules, comparison between the matched and mismatched nodules from manual annotations is provided in Table 3. Similarly, no significant differences between the two groups were noted. Minor variations were detected in malignancy, where the lower category suggests benign nodules. This implies that medical reports may prioritize clinically relevant information, possibly omitting less impactful findings in patient follow-up and management.

**Table 2:** Number of nodules with matching and mismatching (manual annotation and medical reports) based on diameter.

*Diameter (mm)*	?	≤3	]3,6]	]6,9]	]9,12]	]12,15]	]15,18]	]18,21]	]21,24]	]24,27]	]27,30]	> 30	Total
*Matches*	0	66	250	78	28	12	5	3	4	2	0	2	**450**
*Manual Annotation*	0	128	273	71	16	7	0	0	0	1	0	0	**496**
*Text Report*	25	16	38	4	5	2	2	3	0	1	1	3	**100**

**Table 3:** Number of nodules with matching and mismatching based on characteristics and location.

		*Ranking*					
		1	2	3	4	5	6
*Calcification*	*Matches*	0	0	29	26	38	357
	*Mismatches*	0	3	85	7	53	348
*Internal Structure*	*Matches*	443	3	3	1	—	—
	*Mismatches*	489	1	0	6	—	—
*Lobulation*	*Matches*	256	139	45	10	0	—
	*Mismatches*	335	124	30	4	3	—
*Malignancy*	*Matches*	91	154	133	59	13	—
	*Mismatches*	188	157	115	31	5	—
*Margin*	*Matches*	5	15	52	145	233	—
	*Mismatches*	12	17	48	167	252	—
*Sphericity*	*Matches*	5	70	86	131	158	—
	*Mismatches*	16	100	101	118	161	—
*Spiculation*	*Matches*	341	78	24	4	3	—
	*Mismatches*	394	70	28	4	0	—
*Subtlety*	*Matches*	12	40	90	118	190	—
	*Mismatches*	33	79	117	118	149	—
*Texture*	*Matches*	12	10	33	69	326	—
	*Mismatches*	16	8	35	73	364	—
		***Lobes***					—
		**LUL**	**LLL**	**RUL**	**ML**	***RLL***	—
*Location*	*Matches*	103	101	125	47	74	—
	*Mismatches*	122	92	140	49	93	—

**Fig. 3:**
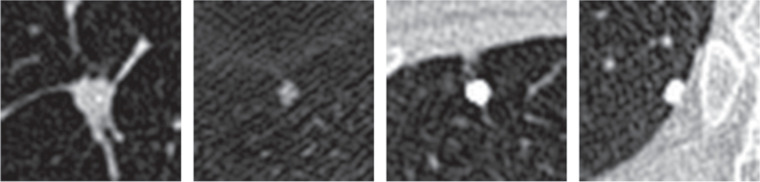
Example of some matched nodules.

**Fig. 4:**
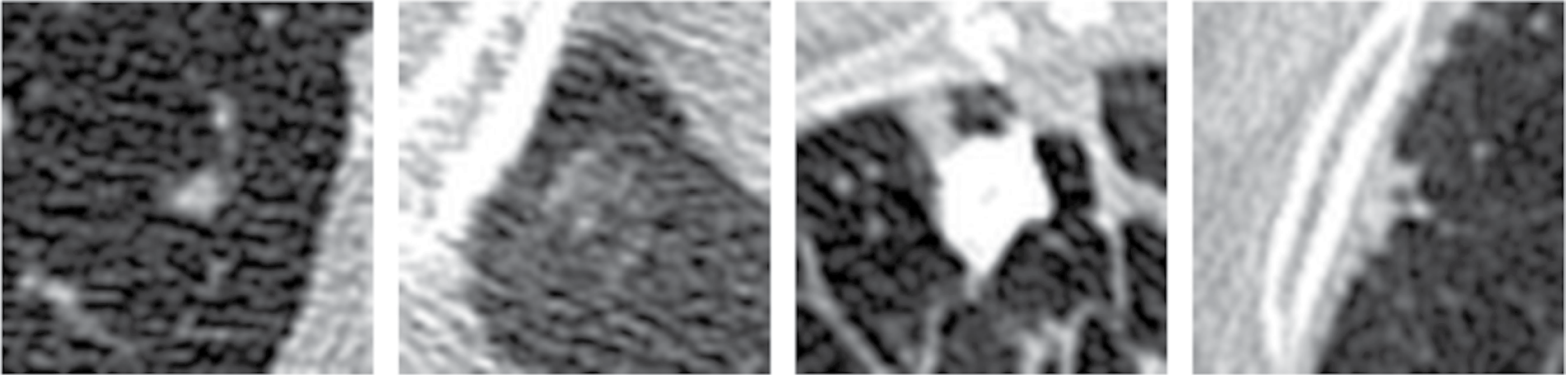
Example of some mismatched nodules.

Figures 3 and 4 display images of the region of interest around the centroid of respectively matched and mismatched nodules. These figures illustrate different types of nodules: isolated, ground-glass opacities (GGOs), juxtapleural, and calcified nodules. There appears to be a noticeable association between the mismatched nodules and surrounding anatomical elements, as well as less typical nodule shapes, which could contribute to their omission in the medical reports.

Finally, for the sets of nodules annotated and identified in the medical reports, the Fleischner score was used, categorizing each exam into four classes: *1)* No routine follow-up required or optional CT at 12 months according to patient risk; *2)* CT at 6-12 months required; *3)* CT at 3-6 months required; and *4)* CT, Positron Emission Tomography (PET), or tissue sampling at 3 months required. The Fleischner score is directly computed using a set of rules that consider the number of nodules (single or multiple), their volume (<100 mm^3^, 100-250 mm^3^, and ≥250 mm^3^), and texture (solid, part-solid, and GGO), as shown in Table 4. A comparison between the manual image annotation and the medical reports can be found in Table 5, using confusion matrices. It is evident that the discrepancy mentioned earlier regarding mismatched nodules does not significantly affect the Fleischner results, as the outcomes are the same for the different sets in 77.1% of cases. This perspective highlights that, in the majority of cases, especially for Fleischner’s follow-up recommendations, the key is to accurately reference the most clinically relevant nodules, and over-annotation and omission of findings may have a slight impact on patient management, as anticipated in the literature.

**Table 4:** Fleischner classification rules used in this study.

Single Nodule	Volume		
	< 100*mm*^3^	100–250*mm*^3^	≥250*mm*^3^
GGO	0	1	1
Part-solid	0	2	2
Solid	0	1	3
**Multiple Nodules**	**Volume**		
	< **100mm**^**3**^	**100**–**250mm**^**3**^	≥**250mm**^**3**^
GGO/Part-solid	2	2	2
Solid	0	2	2
Mixed	Classify for GGO/part-solid and solid nodules independently and give the highest class

**Table 5:** Confusion matrix comparing the Fleischner guidelines’ follow-up recommendation considering the pool of nodules from manual annotations and those mentioned in medical reports.

		*Medical Reports*				
		1	2	3	4	*Total*
Manual Annotation	1	118	3	11	5	**137**
	2	0	5	0	0	**5**
	3	23	8	43	2	**76**
	4	12	1	2	59	**74**
	*Total*	**153**	**17**	**56**	**66**	**292**

## Usage Notes

This study releases the annotated nodules’ characteristics, the medical reports in tabular format, and the matching of nodules between manual image annotation and medical reports. The code used to determine the matching is made available, along with a *help.txt* file and an *environment.yml* to assist potential interested parties in understanding the different parameters used and installing relevant packages.

This publication offers extensive avenues for further research in lung cancer. Instead of solely focusing on comparing detection performance, which may target nodules of little clinical significance, it prompts a reassessment of the relevance of such comparisons and emphasizes the importance of evaluating clinical follow-up recommendations. Additionally, it facilitates the comparison of detection algorithm performance between manually image annotated nodules and those derived from medical reports.

## Data Availability

To assist users in accessing this dataset, we launch a dedicated repository. The code is available on Github (https://github.com/carlosalexnflve/lndb_medicalreports2annotation.git). This repository serves as a central hub for accessing the code and information related to the dataset. It offers users a convenient way to understand the process of matching lung nodules between medical reports and manual image annotation.
